# Lipid Droplets in Cancer: From Composition and Role to Imaging and Therapeutics

**DOI:** 10.3390/molecules27030991

**Published:** 2022-02-01

**Authors:** Patrícia Antunes, Adriana Cruz, José Barbosa, Vasco D. B. Bonifácio, Sandra N. Pinto

**Affiliations:** 1 iBB-Institute for Bioengineering and Biosciences, Instituto Superior Técnico, Universidade de Lisboa, Av. Rovisco Pais, 1049-001 Lisboa, Portugal; patricia.antunes@tecnico.ulisboa.pt (P.A.); adriana.b.cruz@tecnico.ulisboa.pt (A.C.); jose.m.a.b.1996@hotmail.com (J.B.); 2 Associate Laboratory i4HB-Institute for Health and Bioeconomy, Instituto Superior Técnico, Universidade de Lisboa, Av. Rovisco Pais, 1049-001 Lisboa, Portugal; 3 Bioengineering Department, Instituto Superior Técnico, Universidade de Lisboa, Av. Rovisco Pais, 1049-001 Lisboa, Portugal

**Keywords:** cancer cells, chemoresistance, lipid droplets (LDs), LDs imaging, LDs targeting

## Abstract

Cancer is the second most common cause of death worldwide, having its origin in the abnormal growth of cells. Available chemotherapeutics still present major drawbacks, usually associated with high toxicity and poor distribution, with only a small fraction of drugs reaching the tumour sites. Thus, it is urgent to develop novel therapeutic strategies. Cancer cells can reprogram their lipid metabolism to sustain uncontrolled proliferation, and, therefore, accumulate a higher amount of lipid droplets (LDs). LDs are cytoplasmic organelles that store neutral lipids and are hypothesized to sequester anti-cancer drugs, leading to reduced efficacy. Thus, the increased biogenesis of LDs in neoplastic conditions makes them suitable targets for anticancer therapy and for the development of new dyes for cancer cells imaging. In recent years, cancer nanotherapeutics offered some exciting possibilities, including improvement tumour detection and eradication. In this review we summarize LDs biogenesis, structure and composition, and highlight their role in cancer theranostics.

## 1. Introduction

**Figure 1:**
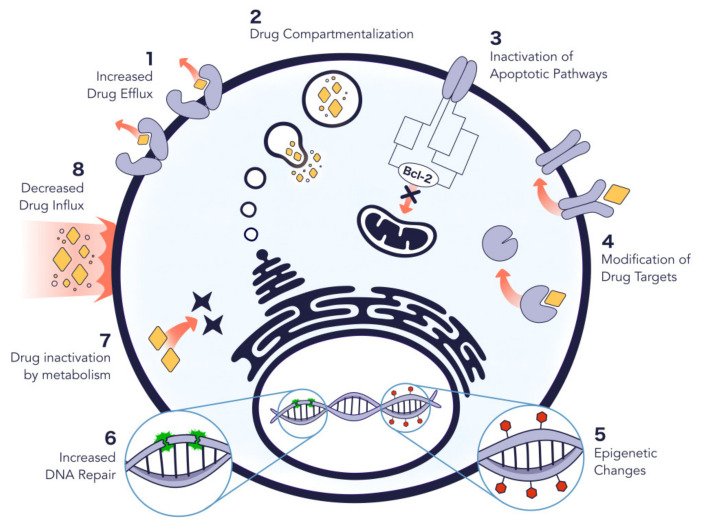
Mechanisms that contribute to the acquired resistance of cancer cells to anticancer drugs. These include increased drug efflux, drug compartmentalization, inactivation of apoptotic pathways, modification of drug targets, epigenetic changes, increased DNA repair, drug inactivation, and decreased drug influx. All these mechanisms have been previously described in detail [9,11].

Cancer is a major public health problem that affects millions of people worldwide. According to WHO’s (World Health Organization) data, cancer is the second leading cause of death globally, with major mortality registered in low- and middle-income countries, and its economic impact has been rising in the recent years [1]. Cancer is caused by an abnormal cellular growth, with the ability to invade other tissues, leading to the formation of tumor masses, neovascularization and metastasis [2,3]. Cancer cells display a negatively charged surface due to a high lactate production [4,5] and altered membrane glycosylation patterns [6], in opposition to healthy mammalian cell membranes, which are mostly zwitterionic [7]. Standard cancer treatments relay on radiotherapy, surgery, and conventional chemotherapy; however, high toxicity is a major drawback, mainly due to insufficient selectivity and unspecific targeting for cancer cells. In addition, another critical issue is drug resistance, either because the initial tumour fails to respond to the treatment or because it acquires resistance during relapse [8]. This resistance may be attributed to the tumor cells genetic instability and heterogeneity, since different genetic backgrounds result in a different reaction to a certain drug [9]. Moreover, the resistance mechanisms can also arise as a response to repeated exposure to cytotoxic drugs, designated by an adaptive response. Several mechanisms can contribute to this acquired resistance including drug inactivation, drug compartmentalization, alteration of drug targets, drug efflux, DNA damage repair, and cell death inhibition (Figure 1) [9]. One of the most studied mechanisms for drug resistance is the increase in drug efflux, which results in a decrease of drug accumulation inside cancer cells [9,10].

Thus, it is fundamental to identify specific molecular biomarkers, common to all cancer cells, and develop novel therapeutic strategies. It is well known that cancer cells can reprogram their glucose and lipid metabolism to survive and proliferate [5,12]. Excess carbohydrates are converted in a faster way into fatty acids. After esterification, leading to triacylglycerols (TAGs) and sterol esters (SEs) [13], these esters are further incorporated into lipid droplets (LDs).

LDs are cytoplasmic organelles composed of core lipid elements, surrounded by an amphipathic lipid layer with several proteins [14,15], and have different diameters, with the range varying from nano- to micron-sizes [16]. Therefore, because of their fast metabolism, cancer cells have a higher content of LDs and LD-related proteins when compared with normal cells. These organelles are produced at the endoplasmic reticulum (ER) and Golgi apparatus, and are constitutively expressed in fat-storing cells, such as adipocytes. Their composition varies according to the type of cancer and the tumor microenvironment [15,17]. Since lipids are crucial for cancer cell proliferation, targeting lipids and their origins might be a powerful strategy to inhibit cancer progression.

Targeted therapy requires the use of a drug that can block and impair the growth of cancer cells. The effectiveness of the therapy relies in the targeted release of the therapeutic agent at the injury site, with greater precision and minimized side effects [18,19]. A targeted delivery system based on nanoparticles has the potential to transform disease management, representing a promising path for cancer therapy [20,21]. The efficient targeting of nanoparticles to the tumor site is currently impacting the development of both therapeutic and diagnostic agents since they can carry chemotherapeutics and/or be used as imaging agents, depending on their properties [21,22,23]. With the progression of nanomedicine, one major goal is the development of nanodevices capable of simultaneously combining therapy and diagnosis (theranostics), allowing real-time monitoring of the treatment progress and efficacy. This approach enables a personalized medicine, directing the treatment for each patient.

Here, we will review LDs biogenesis, growth, and composition, and its role in cancer, highlighting new strategies to target LDs in cancer cells.

## 2. LDs Significance and Impact

### 2.1. Biogenesis and Growth

**Figure 2:**
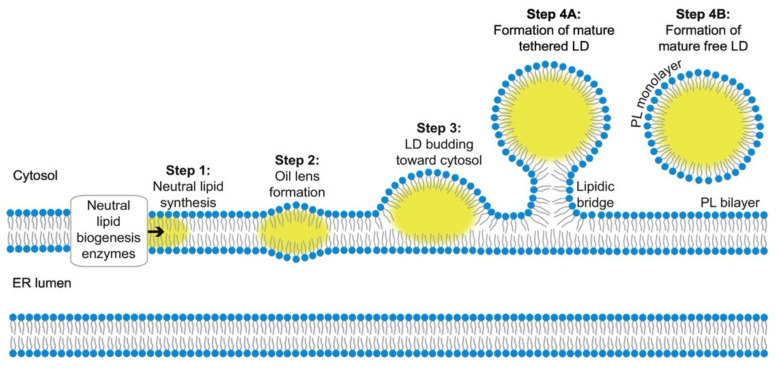
Representation of lipid droplets (LDs) biogenesis and formation. The LDs biogenesis process begins with neutral lipid synthesis in the endoplasmic reticulum (ER), and it is followed by their deposition between the phospholipid (PL) leaflets of the ER bilayer. The neutral lipids stay there until they reach a considerable concentration, and next, they coalesce into oil lenses. The expansion of these nanostructures leads to the budding of LDs from the ER towards the cytosol. The budding process is mediated by FIT proteins and seipin, and it allows the nascent LDs formation. This population of LDs, often called initial LDs (iLDs), will further give rise to expansion LDs (eLDs), which in turn are characterized by having localized TAGs synthesis, allowing their own expansion. [Reprinted with permission from Ref. [41]. Copyright 2020 *BBA Molecular and Cell Biology of Lipids*].

The mechanisms underlying LDs biogenesis are poorly understood; nevertheless, it is known that LDs assembly is a multistep process that occurs in the ER [24]. This organelle provides a hydrophobic environment for fatty acids while allowing contact with the aqueous environment of the lumen and cytosol (Figure 2). There are several proposed models for LDs formation, with the synthesis of neutral lipids being the commonality between them [25]. The synthesis of neutral lipids, generally TAGs and SEs, result from the esterification of fatty acids [24]. This process is accomplished by ER-resident enzymes. SEs are synthetized by acyl-CoA:cholesterol *O*-acyltransferases, ACAT1 and ACAT2, or Are1p and Are2p in yeast; TAGs are made by diacylglycerol acyltransferases, DGAT1 and DGAT2, or Dga1 and Lro1 in yeast [24,25].

It was demonstrated that the synthesis and storage of neutral lipids is not essential for the viability of *Saccharomyces cerevisiae*, since a quadruple mutant lacking these enzymes is viable and lacks LDs [26,27,28]. However, even under optimal laboratory conditions, these mutant yeast cells are very sensitive to stress conditions, such as cell starvation, demonstrating the importance of LDs in cell physiology [24,28]. The recently synthetized neutral lipids are then deposited between the phospholipid leaflets of the ER bilayer, and once they reach a considerable concentration, they coalesce into lenses [29]. These structures are difficult to study since they are likely short-lived and in several cases are very small (30–60 nm in diameter). However, recent studies detected these early lipid droplets intermediates embedded in the ER bilayer [24,30]. The expansion of the oil lenses leads to lipid droplet budding from the ER. In a few cases, LDs have been detected in the ER lumen, though, in most cases, LDs budding occurs towards the cytosol, indicating that the tension in ER membrane is strongly controlled [24]. This budding process is facilitated by some proteins including fat storage-inducing transmembrane (FIT) proteins and seipin [29]. FIT proteins are an evolutionarily conserved group of proteins belonging to the ER membrane [24]. In mammals, there is expression of FIT1 and FIT2 proteins, whilst in other metazoans and yeast there is only expression of FIT2-related proteins [24,31]. FIT1, in mammals, it is primarily expressed in skeletal muscle, and FIT2 is mainly expressed in adipose tissue, suggesting that these two proteins have distinct functions in the organism [32]. Depletion of FIT proteins, in yeast, cause the failure of LDs budding from the ER, and they often become wrapped by the ER membrane [31,33]. Additionally, FIT2 deficiency in adipocytes results in reduced size and number of LDs per cell, indicating that this protein plays an important role in LD biogenesis [32]. However, it is not clear yet how these conserved proteins affect LD budding from the ER membrane.

Seipin, the human Berardinelli–Seip congenital lipodystrophy 2 gene product (BSCL2), is another widely conserved ER membrane protein that is really important for LDs biogenesis [33]. This protein is mainly present at the ER-LD interface, enabling the acquisition of more lipids from the ER and, consequently, the growth of LDs to form mature organelles [29,34]. Depletion of seipin induces the formation of irregular sized LDs (small or super-sized), and eliminates the ability to store fat, causing severe lipodystrophy [25,29]. Without seipin, there is a huge accumulation of nascent LDs in the ER membrane that fail to grow, and the ones that do grow exhibit aberrant enzymes that block the process to form mature LDs [34].

The size of LDs may vary between 0.4 and 100 μm depending on the pathophysiological conditions to which they are subjected [35]. After nascent LDs formation, they can be converted to a subpopulation of LDs, named expanding LDs (eLDs). This leads to the existence of two distinct LDs populations within cells: initial LDs (iLDs) and eLDs. iLDs are formed from the ER, as it was mentioned before, and range from 400–800 nm in diameter [36,37]. This LDs population is further converted into eLDs that are characterized by localized TAGs synthesis allowing their expansion [36]. Although the mechanism behind this process is still unknown, it was found that the Arf1/COPI machinery is involved in this transition, possibly by enabling the establishment of LD-ER bridges that permit the relocation of proteins (such as DGAT2 and GPAT4) from the ER to LDs [37,38]. A possible mechanism to explain the growth of these cytoplasmic organelles is the fusion of highly mobile small LDs that forms organelles with bigger dimensions [39]. Another proposed method is the transfer of the newly synthetized neutral lipids from ER to LDs via Fsp27 (Fat specific protein-27) protein [40]. The expression of this protein forms large LDs through stimulation of neutral lipids accumulation.

### 2.2. Structure and Composition

Once assembled, LDs have a unique structure composed of a hydrophobic core of neutral lipids surrounded by a monolayer phospholipid membrane with several proteins on it. Neutral lipids, mostly TAGs and SEs, are accumulated in LDs at diverse proportions, depending on which cells the organelles are in. For example, in white adipocytes, TAGs are mainly accumulated as lipid esters, whereas in steroidogenic cells, SEs are the most abundant components [35]. Although high resolution images indicate that TAGs and SEs form distinct layers, probably based on their mixing properties, it is not completely understood how neutral lipids’ packaging occurs in droplets [25].

**Figure 3:**
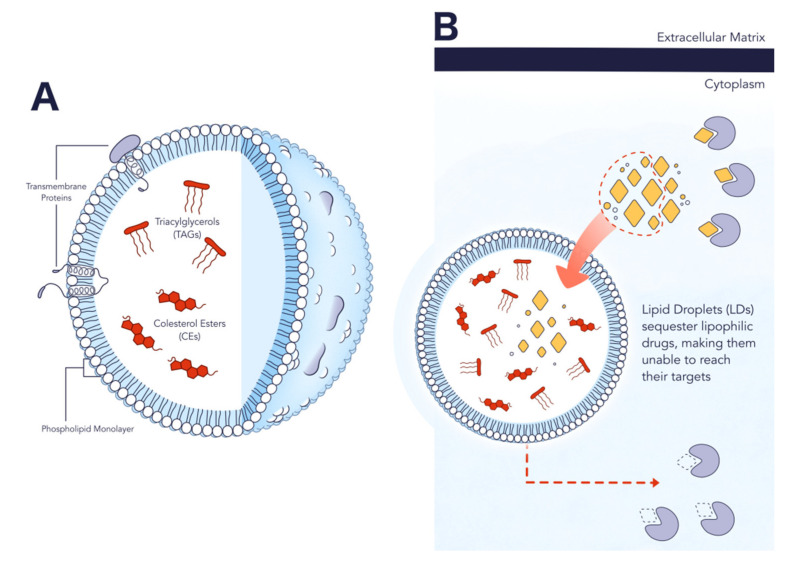
Schematic view of LDs structure, composition, and role in cancer chemoresistance. (**A**) Representation of lipid droplet structure and composition: hydrophobic core composed of neutral lipids (TAG and CEs), phospholipid monolayer, and surface proteins. (**B**) Representation of LDs’s capacity in reducing the efficacy of anti-cancer drugs. LDs offers a compartment to attract and sequester lipophilic compounds, being determinant in cancer chemoresistance.

As LDs develop from the ER membrane, it is expected that the LD phospholipid monolayer presents a similar composition to this membrane. However, some specific enzymes may change this profile during LDs formation, making it different from the original ER membrane [42]. In mammals, phosphatidylcholine (PC) is the main component of the LD monolayer, followed by phosphatidylethanolamine (PE), phosphatidylinositol (PI), phosphatidylserine (PS), sphingomyelin (SM) and lyso forms of PC and PE [35]. In Figure 3, it is possible to observe the molecular structures of the major lipids present on LDs. When there are not sufficient phospholipids to cover the organelles, the surface tension caused by the oil–water interface increases. So, in order to balance this energy cost, LDs tend to fuse, decreasing the overall surface which is covered by more phospholipids [37]. Additionally, alterations in phospholipid ratios under physiological conditions, in several cell types, show that the regulation of the LDs’ monolayer composition is extremely important for the homeostasis of these organelles.

### 2.3. Role in Cancer

Besides the recognized alterations in glucose metabolism, cancer cells are able to reprogram other processes, as is the case with lipid metabolism [12,14,17,43]. This highly proliferative type of cells presents a shift in their lipid metabolism, causing a significant increase in lipid content. They can increase their lipid metabolism by either over activating the endogenous synthesis or the uptake of exogenous lipids and lipoproteins [14].

Huang et al. [44] incubated transformed precursor B cells (TPBCs) in medium containing lipoprotein deficient serum, and they observed a significant growth inhibition and increased cell death. However, when they supplemented the culture medium with very low-density lipoprotein (VLDL), low-density lipoprotein (LDL), or high-density lipoprotein (HDL), the rapid growth of cells was restored. These findings suggested that lipoproteins support tumor growth, and that the proliferation and metastatic capability of cancer cells also depend on lipid metabolism [14].

De novo lipogenesis is the major source of lipids required for the proliferation of carcinogenic cells [12]. Since lipids are crucial for cancer cell proliferation, targeting lipids and their origins may be a useful strategy to inhibit cancer progression. This can be performed by three main approaches: blocking lipid uptake, blocking lipid synthesis, or blocking intracellular lipolysis.

Cancer cells can increase lipid uptake by upregulating cell surface receptors for plasma lipids, such as cluster of differentiation 36 (CD36) [14]. Very recently, it was demonstrated that tumors expressing high levels of CD36 exhibited metastasis potential and that the inhibition of this receptor impairs metastasis [45]. This suggests that blocking lipid uptake is an approach that can be eventually used in cancer treatment.

Regarding lipid synthesis, in many types of cancer, the expression of enzymes responsible for fatty acids synthesis is upregulated. This is the case of acetyl-CoA carboxylase (ACC), fatty acid synthase (FASN), and ATP citrate lyase (ACLY) [14]. The upregulation of enzymes is probably due to the increased expression of sterol-regulatory element binding proteins (SREBPs) in cancer cells. SREBS are transcription factors that activate genes involved in fatty acid and cholesterol biosynthesis [14]. Li et al. found that the inhibition of SREBP impaired cell growth and induced apoptosis in metastatic cancer cells [46]. Targeting lipid metabolism by blocking lipolysis consists of inhibiting the release of LDs content by some enzymes, as is the case of HSL and ATGL. These enzymes, by hydrolyzing lipid droplets, provide a stream of intracellular free fatty acids that stimulate cancer cell proliferation [14]. In this sense, Zagani et al. demonstrated that the knockdown of ATGL inhibits cancer cell growth [47]. In some types of cancer such as glioblastoma multiforme, the highest grade glioma tumor, the synthesis and storage of fatty acids is crucial to cancer cell survival and proliferation [48].

From the above, it is almost clear that there is a positive relation between FAs content and LDs intracellular content. In line with this affirmation, it was reported that the treatment with orlistat, an inhibitor of FASN activity, increased the toxicity of several cancer cell lines and inhibited tumor progression and metastasis (in prostate cancer xenografts and experimental melanomas). The inhibition of FASN in an orlistat-treated mouse caused a decrease in the presence of LDs [49]. Additionally, as described in [50], the addition of oleic acid resulted in a higher content of LDs in HT-29, Hela and MCF-7 cancer cells. Importantly, these cells proved to be more resistant to anti-cancer drugs. This was verified probably because the LDs hydrophobic core offers a compartment able to attract and sequester lipophilic compounds, such as lipophilic drugs [51] (Figure 3B). The entrapment of anticancer drugs makes them unable to reach their targets, which are usually specific genes and proteins in the cell nucleus, reducing their effectiveness [50]. Thus, the inhibition of LDs formation could decrease drug sequestration and, consequently, improve its efficacy. In this context, Zhang et al. [52] tested the treatment of glioblastoma cells treated with curcumin (a promising anticancer agent) in combination with pyrrolidine-2 (RSC-3388), which is an inhibitor of cytosolic phospholipase A2 alpha, a key enzyme in LDs formation. Due to its lipophilic properties, curcumin localizes preferentially in lipid membranes and LDs. Therefore, the authors observed that the inhibition of LDs formation in glioblastoma by pyrrolidine 2 enhances the therapeutic effect of curcumin by decreasing its sequestration in LDs. Likewise, in colorectal cancer, the production of LDs mediated by lysophosphatidylcholine acyltransferase 2 (LPCAT2) was correlated with resistance to 5-fluorouracil and oxaliplatin both in vitro and in vivo [53]. In addition to the above in vivo data, LDs are also associated with tumor aggressiveness [54], and tumorigenic proteins such as phosphatidylinositol-4,5-bisphosphate 3-kinase (PI3K) were shown to be potentially accumulated in cancer cells LDs [55]. Moreover, Bai et al. performed a study that allowed the identification of prognostic LD-associated genes in pancreatic cancer [56]. After analyzing 179 pancreatic cancer samples and 171 normal pancreatic samples with the use of bioinformatic tools, the authors found nine prognostic LD-associated factors in pancreatic cancer. Geng et al. have also been working to establish the LDs role in clinically relevant samples, such as glioblastoma [57]. They reported that tumor tissue samples from glioblastoma patients showed a large content of LDs.

## 3. LDs in Lipid Metabolism-Related Disease Theranostics

### 3.1. Imaging Strategies

**Figure 4:**
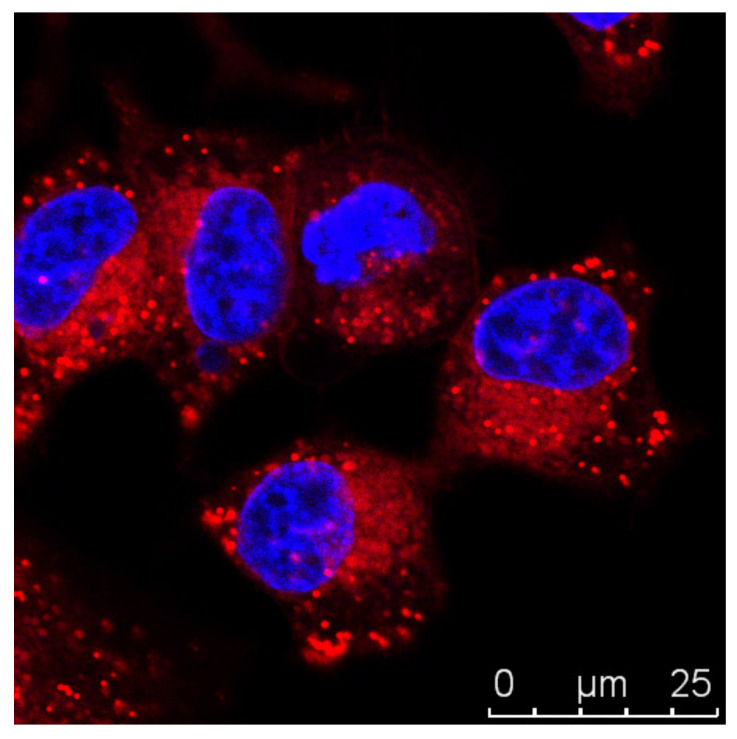
Visualization *of* lipid droplets (LDs) with Nile Red staining. A representative fluorescence confocal microscopy image of live MCF7 cells (human breast cancer cells) was taken using a double staining of Hoechst 33342 (blue), nucleus marker, and Nile Red (red), LDs marker, dyes. Nile Red staining allows the discrimination of LD patterns, seen as spherical structures within the cells.

Since LDs are found to be involved in metabolic diseases, including diabetes, obesity, and cancer, it is crucial to have reliable imaging tools for these organelles. Cellular LDs can be visualized through label-free imaging techniques, as is the case of mass spectrometry and transmission light microscopy, or through fluorescence imaging techniques. The first ones require complex sample preparations, and they usually require the fixation of the cells, or LDs extraction, making the study of the dynamics of LDs within the cell challenging [58]. Fluorescence imaging techniques are very useful to study biological processes, since they are highly sensitive. Nile Red, for instance, is a fluorogenic dye that is used to stain intracellular LDs [59] (Figure 4, unpublished data). Its fluorescence is notorious in most organic solvents and lipidic environments, but very low in aqueous media [59]. However, despite being an easy-to-handle marker, Nile Red presents some disadvantages, including the nonspecific labelling of other lipidic organelles, as is the case of lysosomes [60].

More recently, BODIPY (boron dipyrromethene) dyes were shown to be more selective to LDs than Nile Red, due to better cell permeation. Although BODIPYs are reliable tools for LD staining, they also present disadvantages, such as limited photostability and cross-talk [60].

Regarding fluorescent probes and more complex techniques for LDs imaging, there is still a lack of information (e.g., composition, structure and biogenesis) about these lipidic structures. As described above, different approaches have been developed to monitor LDs inside the cells, such as Raman [58], immunohistochemistry of LDs proteins [61] and fluorescence imaging [59,62,63,64].

Focusing on fluorescence microscopy/techniques to access LDs imaging, some work has been reported in the design of new probes. Donor-acceptor fluorophores, able to promote internal charge transfer (ICT), have been applied in FRET and in super-resolution STED microscopy [65,66,67]. In addition, new dyes such as pyridyl- and thienyl-substituted phospholes, fluorescent fluoranthenes, and solvatochromic coumarins were developed in recent years for LD imaging in living cells and in human cervical cancer tissues [68,69,70]. When compared to other LD staining dyes, such as BODIPYs and Nile Red, these derivatives possess improved optical properties, including larger stoke shifts.

Recently, a study reported the development of a modular fluorophore platform based on boronic acid salicylidenehydrazone-BASHY dyes. The synthesis relies on the condensation of boronic acids (BAs) with a salicylidenehydrazone (SHY) ligand system, leading to dyes with interesting photophysical properties and applications in bioimaging. The authors tested the capability of these dyes for selectively staining LDs in HeLa cells and observed staining without cell viability impairing. Therefore, BASHYs may be a powerful tool for bioimaging applications [71].

Despise these advances, many organic probes still suffer several drawbacks, including poor water solubility, nonspecific staining, and shorter time of intracellular retention [72]. In this sense, the development of nanostructures to act as imaging or delivery imaging agents to LDs may help to overcome these problems. For instance, Klymechenko et al. [73] developed lipid-core nanostructures called nano-droplets, where lipophilic dyes can be loaded at high concentrations. The main advantages of nano-droplets rely on solubilization of dyes in their core, allowing the preservation of the optical properties such absorption/emission and fluorescence lifetime. In this work, 3-alkoxyflavone (F888) and a Nile Red derivative (NR668) were loaded into nano-droplets, and the delivery was found to occur under in vitro conditions.

Quantum-dots (QDs), which are colloidal semiconductor fluorescent nanoparticles, were also successfully applied to the visualization and monitoring of LDs. These probes present several important properties like large stoke shifts (avoiding self-absorption), high photostability, and a narrow and tunable emission window [74,75]. For instance, fluorescence image snaps of lipophilic zwitterionic LQDs (QD-based nanoprobe for lipid droplets)-labeled lipid droplets are visible for minutes after continues irradiation, and LQDs fluorescence does not bleach. Besides that, LQDs enter into the cells by lipid-raft endocytosis and specifically accumulates in LDs [76].

Carbon nanoparticles (CNPs) were also shown to be valuable LDs imaging tools. These nanoparticles show excellent properties, such as water solubility, biocompatibility, and photostability. Liu et al. [72] developed a one-pot synthesis of CNPs based on *o*-phenylenediemine (oPD) and carbon dots (CDs). To ensure staining specificity, co-localization studies were performed using Nile Red as the control. This strategy allowed tracking lipid structures in vitro through time, up to six passages, thus contributing to the development of lipid metabolism-related disease theranostics.

### 3.2. Targeting Novel Therapeutics

As a marker of dysregulated lipid metabolism in cancer cells, LDs might be useful therapeutic targets. One example is photodynamic therapy (PDT), a therapy which is based on the destruction of pathological cells by cytotoxic reactive ROS, generated in a localized region, by the combined action of a ROS photosensitizer (PDT agent) and light irradiation.

In general, photosensitizers (PSs) used in PDT are intrinsically fluorescent and possessing good optical properties. In addition, they might be used on fluorescence-guided treatments (FL-PDT) [77,78,79]. However, conventional PSs, like porphyrins and phthalocyanines, are hydrophobic molecules and can suffer aggregation caused quenching (ACQ) due to π-π interactions in the biological environment [80,81,82]. Besides the ACQ effect in the fluorescence intensity, it is hypothesized that ROS production might also be affected [83,84]. Named as “always on” PSs, these compounds are emissive in non-target tissue which contribute to an increased signal-to-noise ratio. So, to improve FL-PDT efficacy, Jiang et al. developed “turn on” PSs less vulnerable to ACQ effects and more sensitive to target regions. These new PSs, sensitive to aggregation-induced emission (AIE), present excellent optical properties, like large stoke shifts, good photostability, and good environment sensitivity [85,86,87,88]. Furthermore, it is reported that AIE can be related with aggregation-induced ROS generation [89,90,91], which contribute to higher FL-PDT efficacy. Therefore, Jiang et al. developed an amphiphilic pyridinium, TPECNPB, to target LDs in cancer cells by electrostatic interactions. To improve the specificity to cancer cells and LDs, TPECNPB incorporates a boronate ester group that responds to H_2_O_2_ levels of cancer cells in hypoxia [77].

Besides the AIE effect in FL-PDT agents, it is also known that photodynamic theranostics can be improved by promoting the accumulation of the theranostic agent in the cells; therefore, Tabero et al. [92] and others [93,94,95] investigated LDs as possible targets for directing photodynamic theranostics. The capability of LDs to serve as sensitive targets for PDT and photodynamic theranostics was investigated using fluorescent BODIPYs [85], which were found to specifically accumulate in LDs. The success of BODIPYs sparked the interest in the design of novel probes featuring boron derivatives [96].

Other strategies related to new PDT agents included the design of BODIPY-based photosensitizers, BODISeI, with improved single oxygen yield and intersystem crossing [97,98,99]. The ability of BODISeI to target LDs was evaluated in cancer cell lines such as MCF-7 and 4T1 (breast cancer cell lines) and HepG2 (hepatocellular carcinoma). The results showed that BODISeI can damage LDs after light exposure with lower IC_50_ (around 125 nM) [98]. An alternative approach is the development of PDT nanosystems. Dai et al. reported the development of near-infrared AIE nanoparticles (TTI), which are self-assembled nanoparticles with extremely high-efficient singlet oxygen generation efficiency, to target LDs in HepG2 cells (liver hepatocellular carcinoma) [100]. ROS production was confirmed as the main mechanism of action and thus TTI self-assembled nanoparticles can be seen as promising nano-photosensitizers [100]. Likewise, it was demonstrated that some nanoparticles can damage cellular membranes [101,102], thus being a valuable strategy for the development of anti-LDs therapeutics.

## 4. Conclusions and Future Prospects

Cancer is a complex disease that results from abnormal cell proliferation. Anti-cancer therapies rely on surgery, radiation, and chemotherapy. However, these treatments still present major unsolved problems, namely the lack of specificity. Thus, it is urgent to develop novel therapeutic strategies. It is well known that cancer cells can reprogram their lipid metabolism to sustain uncontrolled proliferation. They present a shift in their lipid metabolism and, consequently, a higher production of lipid droplets (LDs). LDs are recognized as important cellular organelles with roles in the lipid and energy homeostasis, in the communication between distinct organelles and possible contribution to other vital cellular processes such as protein degradation. These nano- to micro-sized organelles are surrounded by a phospholipid monolayer also containing diverse neutral lipids (e.g., cholesterol esters) and proteins. Importantly, LDs accumulation is now recognized as a key feature of cancer cells and thus it is crucial to develop imaging probes that allow LDs discrimination in cancer cells, as well as therapeutics that could promote LDs targeting and cancer treatment. In this sense, nanoparticles, for instance, can be viewed as promising systems to track LDs. In fact, the potential of nanoparticles in novel LDs based anti-cancer therapy was recently shown [93]. However, research concerning LDs as a possible hallmark of cancer cells is still very recent, and this review aims at promoting further developments in cancer LD-based theranostics.

## Data Availability

Not applicable.
